# Modeling Inflammation in Zebrafish for the Development of Anti-inflammatory Drugs

**DOI:** 10.3389/fcell.2020.620984

**Published:** 2021-01-15

**Authors:** Yufei Xie, Annemarie H. Meijer, Marcel J. M. Schaaf

**Affiliations:** Institute of Biology Leiden, Leiden University, Leiden, Netherlands

**Keywords:** inflammatory models, tail wounding, chemical-induced inflammation, mutation, drug screen, glucocorticoids

## Abstract

Dysregulation of the inflammatory response in humans can lead to various inflammatory diseases, like asthma and rheumatoid arthritis. The innate branch of the immune system, including macrophage and neutrophil functions, plays a critical role in all inflammatory diseases. This part of the immune system is well-conserved between humans and the zebrafish, which has emerged as a powerful animal model for inflammation, because it offers the possibility to image and study inflammatory responses *in vivo* at the early life stages. This review focuses on different inflammation models established in zebrafish, and how they are being used for the development of novel anti-inflammatory drugs. The most commonly used model is the tail fin amputation model, in which part of the tail fin of a zebrafish larva is clipped. This model has been used to study fundamental aspects of the inflammatory response, like the role of specific signaling pathways, the migration of leukocytes, and the interaction between different immune cells, and has also been used to screen libraries of natural compounds, approved drugs, and well-characterized pathway inhibitors. In other models the inflammation is induced by chemical treatment, such as lipopolysaccharide (LPS), leukotriene B4 (LTB4), and copper, and some chemical-induced models, such as treatment with trinitrobenzene sulfonic acid (TNBS), specifically model inflammation in the gastro-intestinal tract. Two mutant zebrafish lines, carrying a mutation in the hepatocyte growth factor activator inhibitor 1a gene (*hai1a*) and the cdp-diacylglycerolinositol 3-phosphatidyltransferase (*cdipt*) gene, show an inflammatory phenotype, and they provide interesting model systems for studying inflammation. These zebrafish inflammation models are often used to study the anti-inflammatory effects of glucocorticoids, to increase our understanding of the mechanism of action of this class of drugs and to develop novel glucocorticoid drugs. In this review, an overview is provided of the available inflammation models in zebrafish, and how they are used to unravel molecular mechanisms underlying the inflammatory response and to screen for novel anti-inflammatory drugs.

## Introduction

### Inflammation and Inflammatory Diseases

When the body encounters harmful stimuli, such as invading pathogens, wounding or damaged cells, the immune system will be activated and an inflammatory response is triggered (Netea et al., 2017; Chen et al., 2018). This response is induced by Pattern Recognition Receptors (PRRs) such as Toll-Like Receptors (TLRs) recognizing patterns in molecules characteristic for microbes [Pathogen-Associated Molecular Patterns (PAMPs)], or molecules released by damaged cells [Damage-Associated Molecular Patterns (DAMPs)]. Subsequently, immune cells release pro-inflammatory cytokines, such as IL-1β and TNF-α, which in turn stimulate the synthesis and release of inflammatory mediators, including chemokines and prostaglandins (Takeuchi and Akira, 2010; Netea et al., 2017). Directed by the chemokine gradients, leukocytes migrate toward the inflamed site to deal with the damaged tissue or invading microbes (Bonecchi et al., 2009; MacLeod and Mansbridge, 2016). These changes at the molecular level will lead to the five classical symptoms of inflammation: heat, pain, redness, swelling, and eventually loss of function. Normally, the inflammatory processes are actively terminated through functional reprogramming of involved cells, which results in restored homeostasis (Netea et al., 2017).

A dysregulated inflammatory response is observed in various diseases. Abnormally and excessively activated inflammation plays an essential role in the pathogenesis of inflammatory disorders such as asthma, rheumatoid arthritis, and allergic and autoimmune diseases (Marrack et al., 2001; Ngoc et al., 2005). Chronic inflammation in the gastrointestinal tract can lead to inflammatory bowel disease (IBD), which may even cause non-digestive tract complications (Hanauer, 2006). In addition, it has become apparent that chronic inflammation is involved in some diseases that were previously not considered to be inflammation-related, including cancer, type 2 diabetes, neurodegenerative diseases, and atherosclerosis (deLegge and Smoke, 2008; Grivennikov et al., 2010; Mathis and Shoelson, 2011; Geovanini and Libby, 2018). Finally, although inflammation serves primarily as a beneficial defense response against infections, acute or chronic overactivation of the inflammatory response is well-known to exacerbate infectious disease pathologies, for example in COVID-19 and tuberculosis (Kaufmann and Dorhoi, 2013; Merad and Martin, 2020).

Traditionally, the therapeutic regimen for inflammation includes the use of steroidal [glucocorticoid (GC)] and non-steroidal anti-inflammatory drugs (Li et al., 2017). However, the use of these drugs may provoke multiple side effects including osteoporosis, gastrointestinal disorders, cardiovascular or cerebrovascular events, and infection (Antman et al., 2007; Moghadam-Kia and Werth, 2010). Moreover, drug resistance may occur in a subpopulation of patients (Moghadam-Kia and Werth, 2010). In the past decades, successful application of monoclonal antibodies against targets such as TNF-α, CD20, or the IL-6 Receptor, have dramatically improved the prognosis of patients with inflammatory disorders, in particular rheumatoid arthritis (Senolt, 2019). In addition, novel inhibitors of inflammatory signaling pathways involving NF-κB, p38 MAP kinase, T lymphocyte activation, and JAK/STAT have been discovered (O'Neill, 2006; Li et al., 2017). Despite this notable progress, there is still an unmet need for more effective and safer anti-inflammatory drugs. In this review, we discuss the usefulness of the zebrafish as an animal model for studying the mechanims of inflammation and as a screening system to accerelate research aimed at the discovery of novel anti-inflammatory drugs (an overview is presented in Tables 1, 2).

**Table 1:** Overview of zebrafish models for inflammation.

**Inflammatory models**	**Age**	**Treatment**	**Inflammatory responses**	**References**
**Tail wounding-induced inflammation**				
Transection	2–5 dpf	Amputation	Accumulation of macrophages and neutrophils; increased ROS production; upregulated inflammatory genes	Renshaw et al., 2006; Niethammer et al., 2009; Yoo et al., 2011; Enyedi and Niethammer, 2013
	2–4 dpf	Incision	Accumulation of macrophages and neutrophils; increased ROS production;	Mathias et al., 2006, 2009; Enyedi and Niethammer, 2013
Laser	4 dpf	Epidermis	Accumulation of neutrophils	Feng et al., 2010
	22 hpf	Yolk sac	Accumulation of macrophages	Redd et al., 2006; Mathias et al., 2009
	1–2 dpf	Skeletal muscle	Myofibril damage	Otten and Abdelilah-Seyfried, 2013
	2–3 dpf	Tail fin	Accumulation of macrophages and neutrophils; ROS signaling; upregulated inflammatory genes (*tnfa*)	LeBert et al., 2018; Miskolci et al., 2019
**Chemical-induced inflammation**				
LPS	1–3 dpf	Immersion	Increased ROS and NO production; upregulated inflammatory genes (*il1b, tnfa, il10, p65, nfkbia*)	Watzke et al., 2007; Ko et al., 2017
	3 dpf	Yolk injeciton	Accumulation of macrophages and neutrophils; upregulated inflammatory genes (*il1b, tnfa, il6*)	Yang et al., 2014
CuSO_4_	2–7 dpf	Immersion	Hair cell death; infiltration of macrophages and neutrophils; oxidative stress	Hernández et al., 2006; Olivari et al., 2008; d'Alençon et al., 2010; Leite et al., 2013; Carrillo et al., 2016
	Adult	Immersion	Oxidative damage and apoptosis in the gills; upregulated inflammatory genes (*tnfa, mmp9, myd88, il6, il8*)	Craig et al., 2007; Griffitt et al., 2007; Singh et al., 2014
LTB4	3 dpf	Otic vesicle injection	Neutrophil recruitment	Tobin et al., 2010; Deng et al., 2013; de Oliveira et al., 2013
	2 dpf	Hindbrain injection	Macrophage recruitment	Torraca et al., 2015
	3 dpf	Immersion	Neutrophil accumulation in the fin	Yoo et al., 2011; Bischel et al., 2013
Enterocolitis	3–8 dpf	TNBS immersion	Gut dilation; loss of villi and clefts; infiltration of neutrophils; increased number of goblet cells; upregulation of inflammatory genes (*il1b, tnfa, mmp9, ccl20, il8*)	Fleming et al., 2010; Oehlers et al., 2011
	3–6 dpf	DSS immersion	Mucus accumulation; infiltration of neutrophils; reduced proliferation; upregulation of inflammatory genes (*il1b, tnfa, mmp9, ccl20, il8, il23*)	Oehlers et al., 2012
	3–6 dpf	Glafenine immersion	Intestinal epithelial cell apoptosis and shedding; ER stress	Goldsmith et al., 2013; Espenschied et al., 2019
	5–9 dpf	Soybean meal	Infiltration of neutrophils, macrophages and lymphoid cells; upregulation of inflammatory genes (*il1b, tnfa, mpx, saa, c3b, il8*)	Hedrera et al., 2013; Fuentes-Appelgren et al., 2014; Coronado et al., 2019
	Adult	TNBS intrarectal injection	Epithelial disruption; neutrophil infiltration; upregulation of inflammatory genes (*il1b, tnfa,il8, il10*)	Geiger et al., 2013
	Adult	Oxazolone intrarectal injection	Epithelial damage; infiltration of granulocytes; goblet cell depletion; upregulation of inflammatory genes (*il1b, tnfa, il10*)	Brugman et al., 2009
**Mutation-induced inflammation**				
*hai1a*	1–3 dpf		Epidermal defects (skin); leukocyte accumulation; enhanced keratinocytes apoptosis; upregulation of inflammatory genes (*mmp9*)	Carney et al., 2007; Mathias et al., 2007; LeBert et al., 2015
*cdipt*	5–6 dpf		Intestinal damage; reduced mucos ecretion; infiltration of macrophages and neutrophils; globlet cell apoptosis; impaired proliferation; ER stress; upregulation of inflammatory genes	Thakur et al., 2011, 2014

**Table 2:** Overview of drugs showing anti-inflammatory effects in zebrafish inflammation models.

**Inflammatory models**	**Drugs showing anti-inflammatory effect**	**References**
Tail amputation	Extract from *Clerodendrum cyrthophyllum* Turcz	Nguyen et al., 2020
	Extract from *Cymopolia barbata* and its major active component cymopol	Bousquet et al., 2020
	Ginsenoside Rg1 from ginseng; beclomethasone	He et al., 2020
	ErbB kinase inhibitors	Rahman et al., 2019
	A superoxide dismutase from the *Thermus thermophilus* HB27	Sheng et al., 2019
	Extracts from ginseng	Sun et al., 2019
	Bergapten from *Ficus hirta*	Yang et al., 2018
	Meisoindigo, a derivative of indirubin	Ye et al., 2017
	Analogs of thalidomide	Beedie et al., 2016
	*Mircometam C* from *Micromelum falcatum*	Tang et al., 2015
	Isopimpinellin from the Apiaceae family, and other compounds containing a benzopyrone structure	Robertson et al., 2014, 2016
	Tanshinone IIA from *Salvia miltiorrhiza*; dexamethasone	Robertson et al., 2014
	Approved drugs (eg: glipizide, tetracycline HCl, dexamethasone)	Hall et al., 2014b
	5β-Hydroxypalisadin B from *Laurencia snackeyi*; dexamethasone	Wijesinghe et al., 2014
	Compounds from fungal extracts: sterigmatocystin and antibiotic PF1052	Wang et al., 2014
	Fucoidan from *Ecklonia cava*	Lee et al., 2013
Tail incision	Essential oil from Thymus vulgaris	Polednik et al., 2018
LPS immersion	Polyphyllin VII from *Paris polyphylla*	Zhang et al., 2019
	Caffeine	Hwang et al., 2016
	Oleuropein from *Olea europaea*	Ryu et al., 2015
	Polyphenol-rich extract from *Ecklonia cava*	Kim et al., 2014
	5β-Hydroxypalisadin B from *Laurencia snackeyi*; dexamethasone	Wijesinghe et al., 2014
	Fucoidan from *Ecklonia cava*	Lee et al., 2013
LPS injection	A superoxide dismutase from *Thermus thermophilus* HB27	Sheng et al., 2019
	Phillyrin from *Forsythia suspensa Vahl*; dexamethasone	Yang et al., 2017
	Extracts from *Chimonanthus nitens* Oliv.; dexamethasone	Sun et al., 2017
	Chlorogenic acid	Yang et al., 2014
CuSO_4_ immersion	Extract from *Clerodendrum cyrtophyllum* Turcz	Nguyen et al., 2020
	Enzymatic peptide from skipjack (*Katsuwonus pelamis*); indometacin	Wang et al., 2019
	Terpene glycoside from *Sanguisorba officinalis*	Guo et al., 2019
	A superoxide dismutase from *Thermus thermophilus* HB27	Sheng et al., 2019
	Polyphyllin VII from *Paris polyphylla*	Zhang et al., 2019
	Pituitary adenylate cyclase-activating polypeptide (PACAP)-38)	Kasica-Jarosz et al., 2018
	Extract from Quzhou Fructus Aurantii; indometacin	Li et al., 2018
	Clinically approved drugs (eg: tenatoprazole, candesartan)	Wittmann et al., 2015
TNBS immersion	A superoxide dismutase from *Thermus thermophilus* HB27	Sheng et al., 2019
	Cholecystokinin; dopamine receptor agonists; dexamethasone	Oehlers et al., 2017
	5-aminosalicylic acid; prednisolone	Fleming et al., 2010; Oehlers et al., 2011
	NOS inhibitors; thalidomide; parthenolide	Fleming et al., 2010
DSS immersion	Prostaglandin E2, mesalamine, and 6-mercaptopurine	Chuang et al., 2019
	Cholecystokinin; dopamine receptor agonists	Oehlers et al., 2017
	Retinoic acid; dexamethasone	Oehlers et al., 2012

### The Zebrafish as an Animal Model for Biomedical Research

The use of zebrafish (*Danio rerio*) as a research model started in the 1950s and it was initially applied for studying embryonic development (Streisinger et al., 1981). The zebrafish is a tropical fish that grows in freshwater at temperatures between 24.6 and 38.6°C (Engeszer et al., 2007). When zebrafish find a shore of shallow water, they tend to spawn in the morning, which can be easily simulated in the laboratory with sliding bottom inserts and lamp light at 28°C (Eaton and Farley, 1974; Avdesh et al., 2012). The transparent embryonic and larval stages, the relatively short generation time, the small size and strong reproduction ability of zebrafish make it a highly versatile animal model. Over the years, genetic tools and experimental methods have been applied, leading to the successful sequencing of the zebrafish genome, enabling rapid screening of gene function, and the generation of various transgenic or mutant fish lines and models for studying human diseases (Barut and Zon, 2000; Vogel, 2000; Lieschke and Currie, 2007). Due to the accumulation of knowledge and available tools for zebrafish, we are currently able to optimally exploit the advantages of this model.

Although initially used to study embryonic development, the zebrafish has emerged as a versatile animal model in diverse areas of biomedical research, including immunology, toxicology, cancer, and behavioral biology (Tavares and Santos Lopes, 2013; Patton and Tobin, 2019). In recent years, there have been many successful attempts modeling human diseases using zebrafish. For example, the characteristics of benign and malignant tumors that develop in zebrafish are similar to the histological symptoms of human tumors (Amatruda et al., 2002), zebrafish infected with *Mycobacterium marinum* simulate hallmarks of human tuberculosis (Prouty et al., 2003), and the phenotype of zebrafish carrying a mutation in the gene *sauternes* closely resembles the pathology of human X-linked congenital sideroblastic anemia (Brownlie et al., 1998). In this review, we will discuss how the zebrafish is used as an animal model for inflammatory diseases and how the available models have been used for research on anti-inflammatory drugs.

An important advantage of the model is that the small size of zebrafish embryos and the development of automated techniques facilitate high-throughput screening (Carvalho et al., 2011; Meijer and Spaink, 2011; Bischel et al., 2013). Although *C. elegans* and *Drosophila* are also frequently used for high-throughput screening, their cuticles may act as a barrier for diffusion (Strecker et al., 1995; Burns et al., 2010). Zebrafish embryos do not have cuticles, and most drugs can therefore be delivered by simply adding them to the culture medium at a relatively low dose. As a vertebrate, zebrafish are evolutionarily more closely related to mammals compared to worms and flies, so results can be more easily extrapolated to humans. Therefore, zebrafish models have a strong potential to serve as whole animal models to be used in preclinical bioassays during drug development.

In addition to the application of zebrafish for testing the efficacy of drugs in specific disease models, they are also commonly used for testing toxicity of drug candidates. Standardized toxicity tests exist, such as the Fish Embryo Acute Toxicity Test (FET), for which guidelines have been published by the Organization for Economic Co-operation and Development (2013). In addition to these general toxicity assays using mortality and obvious morphological changes as endpoints, more specialized assays are available to determine e.g. reproductive toxicity, hepatotoxicity, nephrotoxicity, cardiotoxicity, and assessment of seizure and drug abuse liability (Miyawaki, 2020; Shen and Zuo, 2020).

The immune system of zebrafish is highly similar to that of humans. The innate branch of the immune system matures first during zebrafish development, and macrophages can be observed from 15 h post-fertilization (hpf) (Herbomel et al., 1999). By the onset of blood circulation at 26 hpf, embryonic macrophages are already capable of phagocytosing particles, producing reactive oxygen species (ROS), and killing pathogens (Herbomel et al., 1999; Hermann et al., 2004). The zebrafish neutrophils, which develop by 18 hpf and mature between 24 and 48 hpf, resemble human neutrophils regarding the segmented nuclei, granules, and expression of myeloperoxidase (Bennett et al., 2001; Lieschke et al., 2001). Additionally, zebrafish show conserved critical parts of the adaptive immune system, including thymus development, thymocyte development and the function of T-cells and B-cells (Langenau and Zon, 2005). The adaptive immune system matures after 3 to 4 weeks (Lam et al., 2004; Page et al., 2013), which means that the innate immune system can be studied separately during early embryonic and larval stages. The inflammatory response has also been found to be well-conserved in zebrafish and this has been successfully exploited to increase our mechanistic understanding of the role of neutrophils in inflammatory diseases (Henry et al., 2013; Shelef et al., 2013). The inflammatory response in zebrafish larvae can be induced using a variety of approaches. In this review we provide an overview of different methods to trigger inflammation (see **Figure 2** for a schematic overview of these different methods), and we discuss how they are used for studies on the molecular mechanisms underlying the inflammatory response as well as for research aiming at the development of novel anti-inflammatory drugs, in particular novel GC drugs.

## Inflammatory Disease Models in Zebrafish

### Wounding-Induced Inflammation

#### Introduction

**Figure 1:**
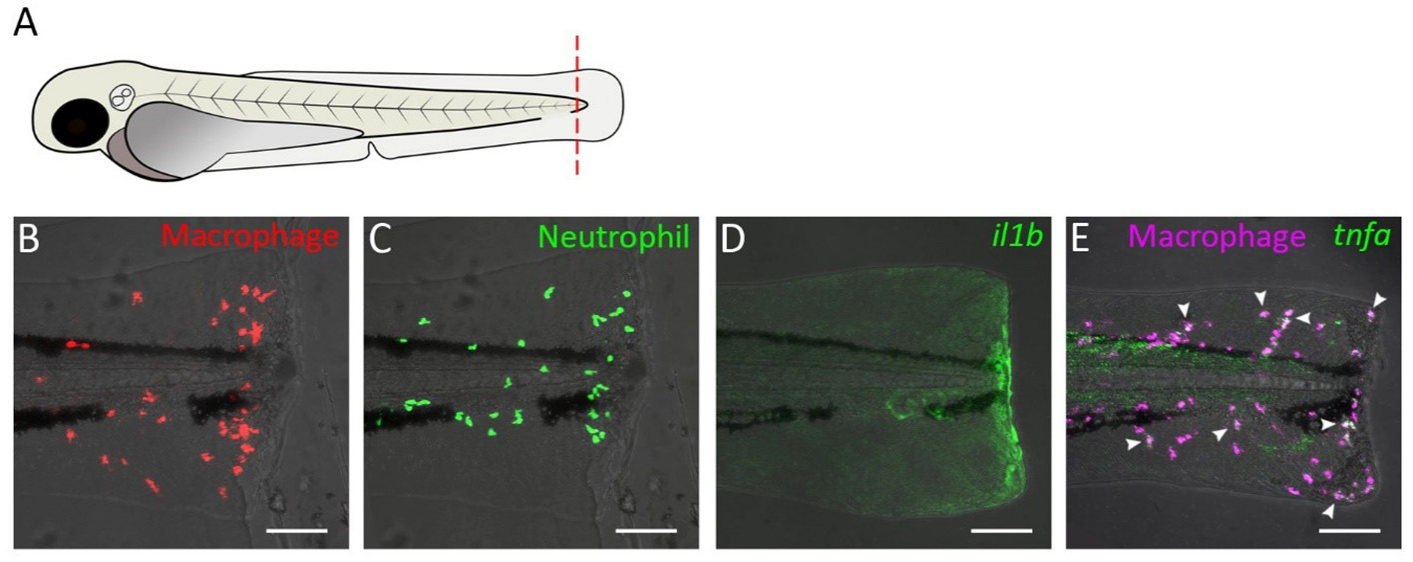
Tail transection in zebrafish larvae as a model for inflammation. **(A)** Schematic drawing of a zebrafish larva at 3 dpf. The dashed red line shows a site of transection (in some studies, the transection site may not include the a part of notochord). **(B–E)** Confocal microscopy images of tail from amputated larvae of the following transgenic lines: *Tg(mpeg1:mcherry-F)* **(B)**, *Tg(mpx:GFP)* **(C)**, *Tg(il1b:GFP)* **(D)**, *Tg(mpeg1:mCherry-F/tnfa:eGFP-F)* **(E)**. Images were taken at 4 h post-amputation using a Nikon Eclipse Ti-E microscope with a Plan Apo 20X/0.75 NA objective. Images show accumulation of macrophage **(B)** and neutrophils **(C)**, activation of the *il1b* gene **(D)**, and *tnfa* expression in macrophage **(E)** near the wound. In **(E)**, arrow heads indicate macrophages in which *tnfa* was activated.

**Figure 2:**
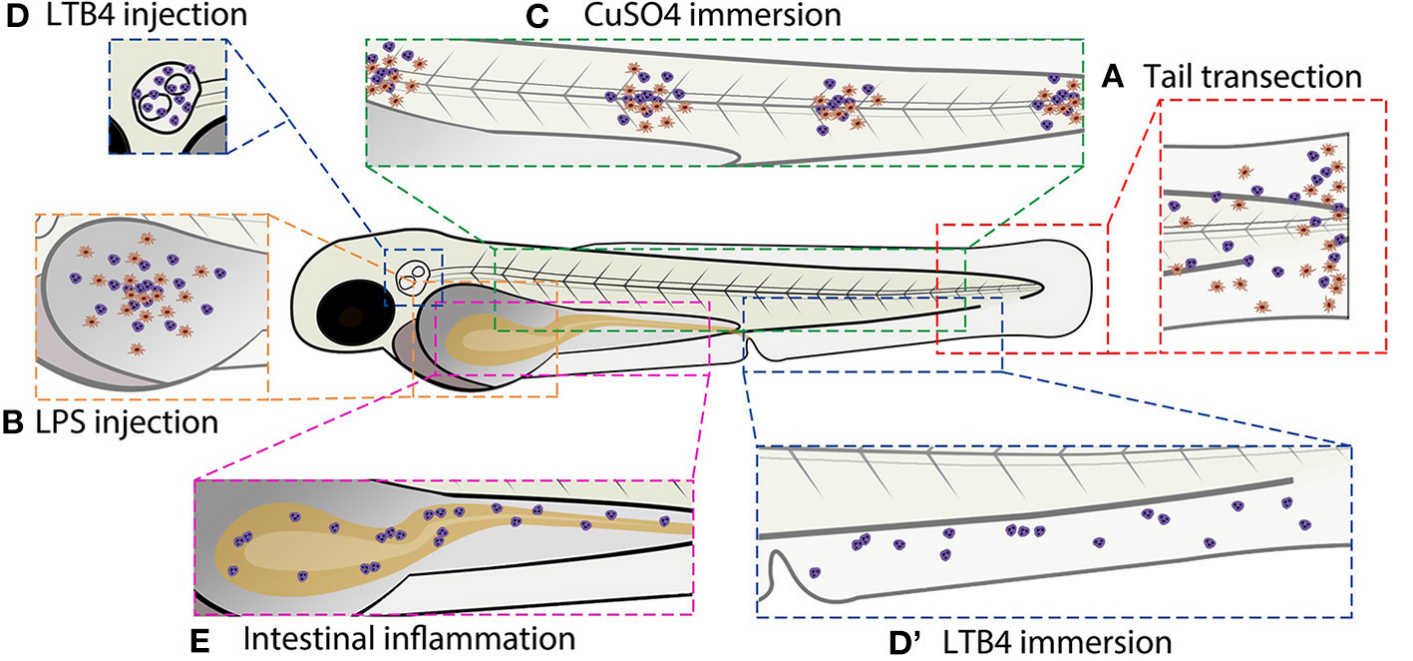
Schematic overview of commonly used zebrafish larval inflammation models. **(A)** Transection of the tail fin. **(B)** LPS injection in the yolk sac. **(C)** CuSO4 immersion causing damage to the neuromasts. **(D)** LTB4 injection in the otic vesicle. **(D′)** LTB4 immersion. **(E)** Chemical-induced intestinal inflammation. All presented models induce leukocyte recruitment. Red cells represent macrophages and purple cells represent neutrophils (in some models, the macrophage infiltration is not shown because it has not been investigated in the studies cited in this review). Alternative zebrafish larval inflammation models, discussed in the text but not presented in this figure, include laser wounding-, tail fin incision-, LPS immersion-, and mutation-induced inflammation. For the tail fin transection/incision, CuSO4 immersion and intestinal inflammation models, adult zebrafish have been used as well.

Acute inflammation induced by tail wounding is a well-established model for inflammation and regeneration studies in zebrafish (Figures 1, 2A). Tail wounding can be performed by amputation of part of the tail fin, or incision of the fin with a sterile scalpel or needle under a stereo microscope, which can be performed in zebrafish embryos, larvae and adults (Lee et al., 2005; Mathias et al., 2006; Renshaw et al., 2006). In embryos (stages up to 72 hpf) and larvae (72 hpf onwards), the amputation may include a distal part of the notochord, to induce a stronger response (Figure 1A). Subsequently, an acute local inflammatory response can be observed, inducing accumulation of macrophages and neutrophils near the wounded area (Renshaw et al., 2006). The visualization of leukocytes is possible through the use of transgenic fish in which the expression of autofluorescent proteins, such as GFP and mCherry, is driven by promoters which are specifically active in neutrophils [such as the myeloperoxidase (*mpx*) (Renshaw et al., 2006) and lysozyme (*lyz*) promoter (Hall et al., 2007)], or in macrophages [such as the macrophage-expressed gene-1 (*mpeg-1*) (Ellett et al., 2011; Bernut et al., 2014) and *mfap4* promoter (Walton et al., 2015)], or by a promoter that marks both these cell types [*coro1a* (Li et al., 2012) and *pu.1* (Peri and Nüsslein-Volhard, 2008; Sieger et al., 2012)]. Besides direct transection, the wounding can also be inflicted by laser irradiation of the epidermis on the trunk (Feng et al., 2010), the yolk sac (Redd et al., 2006), skeletal muscle tissue (Otten and Abdelilah-Seyfried, 2013), or melanocytes over the yolk sac (Mathias et al., 2009) and in the caudal hematopoietic tissue (CHT) (Yoo et al., 2010). Recently, thermal damage inflicted to the tail fin by a cautery pen has been shown to result in a dramatic loss of collagen fibers in the wound region (unlike tail fin transection), which was accompanied by a stronger inflammatory response and a delayed regeneration than observed after tail transection (LeBert et al., 2018; Miskolci et al., 2019). It should be noted that the embryonic and larval tail fins are not a vascularized tissue and that many of the recruited leukocytes migrate from nearby tissues (e.g., the CHT) to the wound. Therefore these models mostly disregard intravascular migration of leukocytes.

#### Studies on Molecular Mechanisms Underlying the Inflammatory Response

Using the zebrafish tail wounding model for inflammation, different molecular pathways of the inflammatory response have been unraveled. As a first response to wounding, the damaged epithelium generates a sustained hydrogen peroxide [H_2_O_2_, a major reactive oxygen species (ROS)] gradient from the wounded site, through local activation of the epithelial NADPH oxidase Duox (Niethammer et al., 2009; Enyedi and Niethammer, 2013). This gradient initiates the recruitment of leukocytes to the wounded area, in particular neutrophils, which use the Src family kinase Lyn as a redox sensor to detect the H_2_O_2_ gradient (Yoo et al., 2011). In addition, epithelial cells have been shown to use fatty acid β-oxidation to increase their mitochondrial ROS production in response to wounding. This process requires the activity of a zebrafish homolog of the mammalian mitochondrial enzyme, Immunoresponsive gene 1 (IRG1), and was shown to contribute to neutrophil recruitment (Hall et al., 2013, 2014a).

In neutrophils, phosphoinositide 3-kinase (PI3K) was found to mediate migration by inducing actin polymerization and generating membrane protrusions at the leading edge through Rac activation and polarization of F-actin dynamics (in a Rac-independent way), which is required for actomyosin-mediated tail contraction (Yoo et al., 2010). Treatment with the microtubule-destabilizing drug nocadozole impairs neutrophil migration toward wounds, even though this process enhances the polarity of F-actin dynamics (Yoo et al., 2012). SHIP phosphatases limit neutrophil mobility and their migration toward a wound, probably by inhibiting PI3K activity (Lam et al., 2012). Neutrophil migration also appears to require the Wiskott-Aldrich syndrome protein (WASp) for their proper migratory behavior (Cvejic et al., 2008).

Macrophages migrate to a wounded area by extension of pseudopods and they are capable of phagocytosing tissue debris (Mathias et al., 2009). Microtubule disassembly by nocadozole inhibited macrophage migration toward a wound, through global activation of Rho kinase (ROK) and thus a loss of the polarity of ROK activity (Redd et al., 2006). Two distinct subsets of zebrafish macrophages were identified using a *Tg(tnfa:GFP)* reporter line, similar to the differentiation processes that are observed in mammalian macrophages (Mosser and Edwards, 2008; Nguyen-Chi et al., 2015). GFP-positive macrophages could already be observed at 1 hour post-wounding, and they are characterized by a flattened and lobulated morphology, and expression of markers characteristic of classically activated, pro-inflammatory (M1) macrophages. Those GFP-positive macrophages could convert to negative ones, which dominate the population at later stages, showing features of alternatively activated, anti-inflammatory (M2) macrophages.

The migration of leukocytes upon tail wounding is dependent on *de novo* protein synthesis, since treatment with the protein synthesis inhibitor cycloheximide was shown to inhibit the migration of neutrophils and macrophages (Chatzopoulou et al., 2016). Both AP-1- and NF-κB-induced transcription have been shown to be involved and the action of these transcription factor complexes is highly regulated by MAP kinase (MAPK) activity. One class of MAPKs, the c-Jun N-terminal Kinases (JNKs), are involved in the regulation of the AP-1-induced transcription, whereas another class, the p38 MAPKs, appeared not to be alter the function of AP-1. Upon wounding, active JNKs were shown to activate c-Jun, which in turn induces the transcription of *mmp13* in neutrophils, which is required for the migration of these cells (Zhang et al., 2008). This JNK/c-Jun/Mmp13 pathway can be inhibited by Mkp-1 (Zhang et al., 2008). In addition, JNK-mediated c-Jun activation results in an increased expression of the *alox5* gene, encoding the 5-lipoxigenase Alox5, a key enzyme involved in the biosynthesis of leukotrienes, including LTB4 (Liu et al., 2013). This pathway was also shown to be required for neutrophil migration upon tail wounding and could be inhibited by activation of the cannabinoid receptor type 2 (Cnr2) (Liu et al., 2013). NF-κB activation, characterized by p65 phosphorylation, was shown to be dependent on the phosphorylation of another class of MAPKs, the Extracellular signal-regulated kinases (ERKs) (Ren et al., 2018). The activation of this pathway was shown to be dependent on the circadian gene *period1b* (*per1b*), and results in an increased expression of pro-inflammatory molecules like *tnfa, il1b, il6*, and *il8* (Ren et al., 2018).

The cytokine IL-8 (or CXCL8) is known to be a potent chemoattractant for neutrophils in mammalian systems (Huber et al., 1991; Baggiolini and Clark-Lewis, 1992). The zebrafish homologs Cxcl8a (Cxcl8-l1) and Cxcl8b.1 (Cxcl8-l2) have been shown to be upregulated upon tail wounding, mediating neutrophil recruitment through Cxcr2 (Sarris et al., 2012; de Oliveira et al., 2013). The chemokines Ccl2 and Cxcl11aa were demonstrated to be required for the wound-induced migration of macrophages by knocking down the expression of the genes encoding their respective receptors, Ccr2 and Cxcr3.2 (Xie et al., 2019). Suppressing the activation of the cytokine IL-1β (by caspase-1 inhibitors and P2X7 antagonists) resulted in attenuated migration of neutrophils and macrophages (Ogryzko et al., 2014). In addition, knockdown of the gene encoding Il-1β by morpholino treatment was shown to decrease the migration of neutrophils toward the wounded area in two studies (although Il-1β appeared to be dispensable for random basal motility) (Ogryzko et al., 2014; Yan et al., 2014). The migration of macrophages was not affected upon by *il1b* morpholino knockdown in one study (Ogryzko et al., 2014), and decreased in another (Yan et al., 2014). The Il-1β pathway (also involving the adaptor protein MyD88) was shown to act independently of NADPH oxidase-mediated ROS production, since treatment with the NADPH oxidase inhibitor DPI did not affect *il1b* expression levels (and vice versa: *il1b* and *myd88* knockdown did not affect ROS production upon tail wounding) (Yan et al., 2014).

Several hours after the wounding, the response enters the resolution phase, and active Wnt/β-catenin signaling has been suggested to play a role in this transition (Petrie et al., 2014). In the resolution phase of the inflammatory response, neutrophils leave the wounded area (a process called reverse migration) or undergo apoptosis (de Oliveira et al., 2016). The survival of neutrophils is regulated by Serum and Glucocorticoid Regulated Kinase 1 (SGK1), which is an anti-apoptotic protein downstream of the neutrophil survival factor GM-CSF (Burgon et al., 2014). The hypoxia-inducible factor-1α (HIF-1α) has been proven to be a critical factor for the regulation of myeloid cell function in mammals, and the activation of Hif-1α delays the resolution of inflammation in zebrafish by inhibiting neutrophil apoptosis and reverse migration (Elks et al., 2011). The reverse-migrating neutrophils were found to exhibit an activated morphology and to respond normally to a secondary challenge such as a local bacterial infection (Ellett et al., 2015). In a recent study it was shown that the reverse migration of neutrophils from the wound to the secondary bacterial infection locus is slowed down upon Hif-1α activation (Schild et al., 2020). The Cxcl12/Cxcr4 signaling axis plays a role in neutrophil retention and the knockdown of *cxcr4b* and *cxcl12a* or the pharmacological inhibition of this signaling increased the movement of neutrophils away from the wounded area (Isles et al., 2019).

Recently, the commensal microbiota has been shown to modulate innate immunity and the inflammatory response in zebrafish, with different bacterial species having different effects (Rolig et al., 2015). Early exposure of zebrafish to commensal microbes primed neutrophils and induced several genes encoding pro-inflammatory and anti-viral mediators. Upon tail wounding, an increased recruitment of neutrophils was observed in the microbiota-colonized zebrafish (Galindo-Villegas et al., 2012; Kanther et al., 2014). This priming effect of the neutrophils by the commensal microbes appeared to be mediated through the Tlr/Myd88 signaling pathway (Galindo-Villegas et al., 2012), and the induction of the acute phase protein serum amyloid A (Saa) was required for the increased migration of the neutrophils. This effect of Saa was shown to be mediated by NF-κB-dependent gene expression (Kanther et al., 2014). In addition, a short-chain fatty acid produced by microbes in the gut, butyrate, was shown to reduce neutrophil migration to a wound via a Hydroxycarboxylic acid receptor 1(Hcar1)-dependent pathway and to reduce the recruitment of M1 macrophages, independent of Hcar1 (Cholan et al., 2020). For regeneration studies, the amputation is often performed on 2 days post fertilization (dpf) zebrafish, after which tissue repair can be observed gradually and complete regeneration can be established 3 days later, at 5 dpf, which is in many countries (including those belonging to the European Union) within the time frame in which regulations of animal experimentation do not apply (Kawakami et al., 2004; Mathew et al., 2007). It was demonstrated that the tissue regeneration of zebrafish embryos is dependent on ROS-induced vimentin production at the wound edge, and that the Stat3 and Tgfβ signaling pathways are involved in this process (LeBert et al., 2018; Miskolci et al., 2019). Furthermore, It was shown that regeneration was not affected after ablation of macrophages and neutrophils using morpholino knockdown of the *pu.1/spi1b* gene, which encodes a transcription factor required to permit myeloid cell development (Mathew et al., 2007). However in later studies, macrophages were shown to be crucial for cell proliferation and tissue regeneration, since ablation of macrophages by an *irf8* morpholino (which drives myeloid cell fate toward neutrophil development) resulted in impairment of the fin regeneration, and the presence of large vacuoles in the regenerated tissue (Li et al., 2012). A specific subset of macrophages, peripheral tissue-resident macrophages, were shown to contribute to tail fin regeneration by ROS production and downregulation of inflammatory mediators such as Il-1β at the damaged site (Morales and Allende, 2019). In the adult zebrafish tail fin amputation model, macrophages have also been shown to enhance tail fin regeneration, by regulating tissue growth and bone ray patterning, which was demonstrated by depletion of macrophages in transgenic fish using the nitroreductase (NTR)/metronidazole(MTZ) cell ablation technology (Petrie et al., 2014). Mutation of the *runx1* gene reduced neutrophil numbers, but did not affect tail fin regeneration (Li et al., 2012). These findings suggest that the inflammatory response induced by wounding, in particular the recruitment of macrophages, is critical for tissue repair and regeneration.

#### Drug Discovery Studies

Tail wounding-induced inflammation in zebrafish has been used for anti-inflammatory drug testing and screening in numerous studies. Natural compounds, well-characterized drugs and defined pathway inhibitors have been tested and several libraries of such compounds have been screened in this model system. These studies contributed to the validation of this inflammation model and resulted in the identification of a number of novel anti-inflammatory compounds, requiring validation in other models and further optimization and testing.

In order to find new anti-inflammatory drugs from collections of existing or clinically approved drugs [drug repositioning (Ashburn and Thor, 2004)], a library of approved drugs that had not previously been characterized as anti-inflammatory compounds, were screened for their ability of suppressing neutrophil recruitment in the zebrafish tail wounding assay (Hall et al., 2014b). Interestingly, the 10 most potent repositioned drugs from this zebrafish screen (including amodiaquin dihydrochloride, alfuzosin hydrochloride, and clonidine hydrochloride) also displayed anti-inflammatory activity in a mouse model of skin inflammation. To discover novel analogs of an existing drug with reduced side effects, several analogs of thalidomide were screened using the zebrafish tail wounding model. A number of these analogs were shown to cause a reduction in neutrophil recruitment, without displaying the infamous teratogenic side effects of the original drug (Beedie et al., 2016). Important drug targets for accelerating the resolution of inflammation, ErbBs, were identified by screening kinase inhibitors in the zebrafish tail fin wounding model (Rahman et al., 2019). ErbB inhibitors and simultaneous gene knockdown of two genes that encode ErbB kinases (*egfra* and *erbb2*) resulted in suppression of neutrophil apoptosis and reduced the level of inflammation in zebrafish larvae.

In addition, structure-function studies have been performed using this model. For example, meisoindigo, which is a derivative of indirubin, a chemical constitute of the traditional Chinese herbal medicine Qing Dai was found to inhibit leukocyte migration induced by tail wounding without affecting reverse migration or Akt and Erk activity, whereas indirubin (which represents the core structure of meisoindigo) did not show an effect (Ye et al., 2017). Moreover, a particular chemical group, consisting of fused benzene and pyran rings with an attached carbonyl group (1,4-benzopyrone) or its isomer “coumarin” (1-benzopyran-2-one), was found to be present in four of the nine most-active pro-resolution compounds identified in a large screen of 2,000 well-characterized and approved drugs. All four of these drugs accelerated the resolution of the inflammation and three of them also inhibited neutrophil migration toward the wound (Robertson et al., 2014). Other compounds containing this benzopyrone structure were shown to have similar effects and the most active one, isopimpinellin, was found to inhibit the recruitment of leukocytes (by inhibiting PI3K), and to promote the resolution phase (by inducing neutrophil apoptosis) (Robertson et al., 2016).

Natural compound libraries are rich sources for drug discovery. Various natural products have been demonstrated to have an inhibitory effect on the infiltration of leukocytes near the wounded area, including extracts from the medicinal herb ginseng (roots of plants in the genus *Panax, such as Panax ginseng*) (Sun et al., 2019). One of the bioactive compounds in these extracts was shown to be the ginsenoside Rg1, a glycosylated steroid that exerts its anti-inflammatory activity through the glucocorticoid receptor (GR) (He et al., 2020). Using a library of fungal extracts, two compounds, sterigmatocystin and the antibiotic PF1052, were found to inhibit neutrophil recruitment (Wang et al., 2014). Similar effects on leukocyte migration have been observed for an essential oil from *Thymus vulgaris* (Polednik et al., 2018), for the coumarin-derivative bergapten isolated from *Ficus hirta* roots (Yang et al., 2018), for a hyperthermostable superoxide dismutase from the *Thermus thermophilus* HB27 (TtSOD) (Sheng et al., 2019), for an extract from the green seaweed *Cymopolia barbata* and its major active component, cymopol (Bousquet et al., 2020), and for the compound *micrometam C* isolated from *Micromelum falcatum* trees, which are mangrove associates (Tang et al., 2015). Downregulation of the expression of various pro-inflammatory genes and upregulation of the anti-inflammatory gene *il10* in the tail-wounding assay was found for an extract from *Clerodendrum cyrthophyllum* Turcz leaves (Nguyen et al., 2020). Inhibition on tail wound-induced ROS generation was shown for a metabolite isolated from the red seaweed *Laurencia snackeyi*, 5β-Hydroxypalisadin B (Wijesinghe et al., 2014), for bergapten (Yang et al., 2018) and for the polysaccharide fucoidan, extracted from the brown seaweed *Ecklonia cava* (Lee et al., 2013). The latter two compounds also attenuated the inflammatory response by inhibiting the synthesis of Nitric Oxide (NO), which is an important inflammatory mediator. Enhancement of the resolution of the inflammation, by promoting neutrophil apoptosis and reverse migration, was demonstrated for tanshinone IIA, a compound extracted from the root of the plant species *Salvia miltiorrhiza* (Robertson et al., 2014).

### Chemical-Induced Inflammation

#### LPS-Induced Inflammation

Lipopolysaccharide (LPS) is an endotoxin in the cell walls of Gram-negative bacteria which acts as a PAMP that induces the innate immune response upon recognition by TLRs (Beutler and Rietschel, 2003). LPS-induced inflammation in zebrafish is generally established by non-invasive immersion of embryos in egg medium containing LPS (Watzke et al., 2007; Novoa et al., 2009) or injection into the yolk (Yang et al., 2014; Figure 2B). In mammals, the immune response to LPS is characterized by TLR4-mediated induction of NF-κB and the expression of various inflammatory mediators, including TNFα and IL-1 (Chow et al., 1999; Akira and Takeda, 2004). However, the TLR4 paralogs identified in zebrafish do not recognize LPS, due to the differences in extracellular structures for recognition and the lack of essential costimulatory molecules (Iliev et al., 2005; Sepulcre et al., 2009; Sullivan et al., 2009).

Despite the poorly characterized recognition mechanism for LPS, a response similar to that observed in mammalians has been observed, indicating a high degree of conservation between the zebrafish and mammalian transcription factors and signaling pathways in response to LPS stimulation (Copeland et al., 2005; Forn-Cuní et al., 2017). LPS stimulation enhanced the production of NO and ROS, increased the levels of iNos and Cox2 proteins, and the mRNA levels for *p65, nfkbiaa* and other genes encoding key pro-inflammatory cytokines including *tnfa* and *il1b* (Watzke et al., 2007; Ko et al., 2017). Pre-treatment of zebrafish with a sublethal dose of LPS was shown to prevent mortality as a result of a subsequent lethal dose of LPS, which demonstrates that tolerance, generally observed in mammals, can be reproduced in zebrafish. Cxcr4 signaling appeared to play an important role in the occurrence of LPS tolerance (Novoa et al., 2009; Dios et al., 2014).

LPS-induced inflammation in zebrafish has been used as a model for research on anti-inflammatory drugs. A lot of compounds and extracts from traditional medicinal or non-medicinal herbs were tested using this model, and several of these showed inhibition on LPS injection-induced inflammation. Chlorogenic acid, a polyphenolic compound which occurs in coffee and phillyrin (the main ingredient in *Forsythia suspensa* Vahl fruits) inhibited macrophage and neutrophil recruitment to the site where LPS was injected, and improved the survival rate (Yang et al., 2014, 2017). The latter compound inhibited the MyD88/NF-κB signaling pathway by decreasing expression levels of genes encoding IκBα, Il-1β, Il-6, and Tnf-α (Yang et al., 2017). Extracts from *Chimonanthus nitens* Oliv. leaves also inhibited recruitment of neutrophils (and not macrophages), reduced the LPS-induced upregulation of *il1b, il6*, and *tnfa* expression (Sun et al., 2017). The protein TtSOD, which inhibited tail wounding-induced neutrophil migration, was also shown to inhibit neutrophil infiltration upon LPS injection (Sheng et al., 2019).

In many studies, the ROS and/or NO production have been used as a readout for the anti-inflammatory effect. Polyphyllin VII (PP7) from *Paris polyphylla* inhibited NO generation, and also deceased the heartbeat and attenuated the yolk sac edema after LPS injection into the yolk sac (Zhang et al., 2019). Fucoidan and a polyphenol-rich fraction extracted from *Ecklonia cava* inhibited both NO and ROS formation (Lee et al., 2013; Kim et al., 2014), just like the compound 5β-Hydroxypalisadin B, which was also shown to be anti-inflammatory in the tail-wounding model (Wijesinghe et al., 2014). The polyphenol-rich fraction extracted from *Ecklonia cava* also decreased cell death and improved survival (Kim et al., 2014). In some reports, only the NO generation was used as an indicator for the anti-inflammatory effect of drugs, and this has been used to demonstrate the effects of caffeine (Hwang et al., 2016) and oleuropein, a phenolic compound present in olives and leaves of the olive tree (*Olea europaea*) (Ryu et al., 2015).

Apolipoprotein(apo)A-I is one of the major constituents of high-density lipoproteins (HDLs) which has been shown to have anti-inflammatory effects (McDonald et al., 2003). The role of apoA-I modification was tested in zebrafish embryos by co-injecting LPS and HDLs containing either native or glycated apoA-I. The results demonstrated a reduced mortality upon injection of HDLs with native apoA-I, probably due to its anti-inflammatory effect (Park and Cho, 2011).

LPS treatment has also been used in combination with tail wounding to enhance leukocyte accumulation near the wound. This model was utilized to evaluate the bioactivity of structurally diverse natural products of an East African medicinal plant, *Rhynchosia viscosa*, resulting in the identification of both known and novel isoflavone derivatives with anti-inflammatory activity (Bohni et al., 2013; Cordero-Maldonado et al., 2013).

#### Copper-Induced Inflammation

Copper is a trace element acting as a catalytic cofactor for various enzymes involved in energy and antioxidant metabolism (Linder and Hazegh-Azam, 1996). Excessive inorganic copper from the environment could disturb the copper balance in zebrafish and lead to an inflammatory response mediated by damage from the oxidative stress (Pereira et al., 2016). In adults, soluble copper was reported to induce oxidative damage and apoptosis in the gills and showed dose-dependent lethality (Craig et al., 2007; Griffitt et al., 2007). Upon copper sulfate (CuSO_4_) treatment, the neutrophils in the kidney marrow were found to be activated, and analysis of the proteome of neutrophils revealed regulation of proteins involved in cell cycle, NO signaling, regulation of cytoskeleton, and immune-related processes (Singh et al., 2014).

Exposure of zebrafish embryos to CuSO_4_ was reported to inhibit the survival and development of embryos (Dave and Xiu, 1991; Johnson et al., 2007). It induces an inflammatory status, which is related to exacerbated damage and oxidative stress, and the endogenous signaling molecule adenosine was shown to be involved (Leite et al., 2013). Importantly, within 2 h this treatment induces death of hair cells in the neuromasts of the lateral line, which regenerate and reach full functionality 1 day later (Hernández et al., 2006; Olivari et al., 2008). This damage to the neuromasts results in a localized robust inflammatory response in the neuromasts, including the infiltration of macrophages and neutrophils (d'Alençon et al., 2010; Figure 2C). The recruited macrophages play a critical role in the regeneration of damaged hair cells since ablation of macrophages significantly delays this process, while neutrophils are not required (Carrillo et al., 2016).

The accumulation of neutrophils in the neuromasts is one of the most frequently used indicators for the level of inflammation in this model and has been applied to assess the effect of known anti-inflammatory drugs (d'Alençon et al., 2010). Since the induction of inflammation by CuSO_4_ can be established by just adding the compound into the culture medium, an automated high-throughput drug screening assay could be developed with this model based on leukocyte accumulation around neuromasts, using a double transgenic line with the neutrophils labeled in red and the neuromasts in green [using the claudin b (*cldnb*) promoter driving GFP expression] (d'Alençon et al., 2010; Wittmann et al., 2012). Using this automated system, various drugs from a library of clinically approved drugs were identified to have an anti-inflammatory effect, among which the NOS1 inhibitor 3-Bromo-7-nitroindazole. Further investigation revealed a novel pro-inflammatory role of NO signaling via soluble guanylate cyclase and in a soluble guanylate cyclase—independent manner through protein S-nitrosylation (Wittmann et al., 2015).

Furthermore, a neuropeptide, pituitary adenylate cyclase-activating polypeptide(PACAP)-38, known to be an anti-apoptotic and anti-inflammatory factor, was reported to inhibit neutrophil migration toward the neuromasts and expression of pro-inflammatory genes (*il8, il1b, il6*, and *atf3*) (Kasica-Jarosz et al., 2018). Several natural products were reported to exert an inhibitory effect on the CuSO_4_-induced neutrophil accumulation, including a new terpene glycoside extracted from the root of *Sanguisorba officinalis* (Guo et al., 2019), an enzymatic peptide (SEP) from skipjack (*Katsuwonus pelamis*) (Wang et al., 2019) and an extract from Quzhou Fructus Aurantii, an unripe fruit from the bitter orange tree (*Rutaceae Citrus changshan-huyou Y. B. Chang)* (Li et al., 2018). The compound PP7 (from *Paris polyphylla)* (Zhang et al., 2019) and TtSOD (Sheng et al., 2019) also showed an inhibition of the neutrophil migration upon CuSO_4_ stimulation, similar to what was observed for these compounds in the LPS-induced inflammation model. An extract from leaves of *Clerodendrum cyrtophyllum* Turcz decreased the oxidative stress induced by CuSO_4_ and inhibited inflammation by downregulating genes related to inflammatory processes (*cox2, pla2, c3a, mpx*) and cytokines (*il1b, il]8, tnfa*, and *il10*) (Nguyen et al., 2020).

#### LTB4-Induced Inflammation

Leukotriene B4 (LTB4) is an eicosanoid released by leukocytes, acting as a pro-inflammatory mediator and enhancing leukocyte accumulation at sites of inflammation (Yokomizo et al., 2001; Peters-Golden et al., 2005). In zebrafish, LTB4 was demonstrated to attract both neutrophils and macrophages (Tobin et al., 2010; Torraca et al., 2015). Upon injection of LTB4 into the otic vesicle of 3 dpf zebrafish larvae, neutrophil recruitment to the ear was observed at 1 h after the injection, and this recruitment was not dependent on Cxcl8/Cxcr2 signaling (Deng et al., 2013; de Oliveira et al., 2013; Figure 2D). In addition, injection of LTB4 into the hindbrain at 30 hpf recruited macrophages independent of Cxcl11aa/Cxcr3.2 signaling (Torraca et al., 2015). Bath application of LTB4 induced dissemination of neutrophils into fins, which can be quantitated by counting cells in the ventral fin (Figure 2D′). This LTB4-induced migration of neutrophils was not prevented by inhibition of the Cxcl8/Cxcr2 signaling pathway either (Deng et al., 2013), or by DPI or Lyn knockdown (Yoo et al., 2011). A Zebrafish Entrapment by Restriction Array (ZEBRA) microfluidic device was designed to quickly position zebrafish embryos and larvae in a predictable array, suitable for automated imaging. The effectiveness of this device was demonstrated by assessing the inhibitory effect of the PI3K inhibitor LY294002 on LTB4-induced neutrophil migration (Bischel et al., 2013). The device can be designed with access ports to enable the administration of treatments, and it could potentially be used for other inflammation assays like tail wounding as well (Bischel et al., 2013).

#### Chemical-Induced Intestinal Inflammation

Inflammatory bowel disease (IBD) represents a group of intestinal disorders that are characterized by inflammation of the digestive tract (Hanauer, 2006). IBD is modeled in zebrafish by treatment of fish with chemicals that induce an IBD-like enterocolitis (Lee and Renshaw, 2017; Figure 2E). In adult zebrafish, intrarectal administration of the hapten oxazolone was reported to induce enterocolitis, characterized by infiltration of granulocytes, epithelial damage, goblet cell depletion, and upregulated expression of genes encoding cytokines (*il1b, tnfa, il-10*) (Brugman et al., 2009). Similar results were obtained upon intrarectal administration in adult zebrafish of another hapten, trinitrobenzene sulfonic acid (TNBS), which was also shown to increase the intestinal mRNA and peptide levels of melanin-concentrating hormone (Mch) and the mRNA levels encoding its receptor (Geiger et al., 2013).

Immersion of larvae in egg water containing TNBS between 3 and 8 dpf induced an inflammatory response in the gut (Fleming et al., 2010). Using a fluorescent dye, which was swallowed by the fish, the gut architecture and motility could be assessed, showing TNBS-induced dilation of the gut, reduction in villus length, expansion of crypts, and a loss of peristalsis. Throughout the length of the gut, histological analysis showed an expansion of the lumen, a smoothening of the gut lining which was devoid of villi and clefts, and an increase in the number of goblet cells (Fleming et al., 2010). The reported changes in intestinal cell morphology were not observed in a different study in which different doses and durations of TNBS exposure were used (Oehlers et al., 2011). In this study an increased number of neutrophils in the inflamed intestine and increased expression of *il1b, tnfa, mmp9, ccl20*, and *il8* was observed upon TNBS treatment (Oehlers et al., 2011).

Exposure of zebrafish larvae to the detergent dextran sodium sulfate (DSS) has also been utilized to induce intestinal inflammation. This treatment recapitulates several aspects of TNBS-induced enterocolitis, inducing symptoms such as elevated expression of pro-inflammatory genes and neutrophil recruitment around the intestine (Oehlers et al., 2012). However, the inflammatory phenotype was not identical to that induced by TNBS, and DSS treatment actually protects against TNBS-induced enterocolitis (Oehlers et al., 2012). The non-steroidal anti-inflammatory drug glafenine has also been used in zebrafish larvae to induce intestinal injury after 12 h of exposure, which is characterized by profound intestine-specific pathological changes. Glafenine was shown to induce intestinal epithelial cell apoptosis and shedding, which resulted from ER stress and the induction of the unfolded protein response (Goldsmith et al., 2013). The inhibition of multidrug resistance (MDR) efflux pumps by glafenine appeared to play an important role in the intestinal epithelial cell shedding. This shedding plays a protective role by restricting inflammation and promoting survival (Espenschied et al., 2019).

In addition, soybean meal, which is commonly used in aquaculture to replacement for fish meal as a protein source in fish food, has been shown to trigger intestinal inflammation in zebrafish larvae. This inflammatory response is characterized by neutrophil recruitment to the intestine and increased transcription levels of genes encoding pro-inflammatory cytokines (*il1b* and *il8*), and was shown to result from soy saponin and not soy protein (Hedrera et al., 2013). In a later study, both soybean saponins and protein isolates were shown to increase the number of neutrophils (stained using Sudan black) in the intestine and the expression of genes related the innate immune system (*il1b, tnfa, mpx, saa and mpx, saa, c3b*) (Fuentes-Appelgren et al., 2014). Furthermore, the inflammation was shown to increase the epithelial permeability, decrease protein absorption and alter the composition of the intestinal microbiota (Solis et al., 2020). The soybean meal-induced intestinal inflammation is dependent on the presence of lymphoid cells present in the intestine, which correspond to T helper cells with a Th17 transcriptional profile (Coronado et al., 2019). When focusing on long-term effects, the early stage feeding of soybean meal or soy protein was found to have programming effects on inflammation at the juvenile stage (Perera and Yufera, 2016). Since soybean meal is an important commercial food source for fish, studies have been done to identify additives for compensating its pro-inflammatory effects and a few candidates were shown to inhibit soybean meal-triggered intestinal inflammation, including lactoferrin (Ulloa et al., 2016), microalgae (Bravo-Tello et al., 2017), aloe vera (Fehrmann-Cartes et al., 2019), the typical cholinesterase inhibitor galantamine (Wu et al., 2020), and a phytase-producing strain of *Bacillus subtilis* (Santos et al., 2019).

Just like in humans suffering from IBD and in mouse models of IBD (Packey and Sartor, 2008), the variable composition of the gut microbiota was demonstrated to be an important determinant of intestinal inflammation in zebrafish [with an exception in soybean meal-induced intestinal inflammation (Solis et al., 2020)]. Treatment of adult zebrafish with vancomycin or colistin sulfate differentially affected the components of the intestinal microbiota, which influenced the severity of the oxazolone-induced enterocolitis and the composition of the intestinal leukocyte infiltration (Brugman et al., 2009). In larvae, treatment with the broad-spectrum antibiotics kanamycin and ampicillin, which resulted in a severe loss of microbiota, decreased mortality after TNBS exposure, and inhibited the induction of pro-inflammatory gene expression and leukocyte migration to the intestine (Oehlers et al., 2011). Using a protocol to generate germ-free zebrafish larvae, it was confirmed that the TNBS-induced pathology, including histological changes and an increased expression of genes encoding pro-inflammatory cytokines, entirely depended on the presence of resident microbiota (He et al., 2014). TNBS-induced enterocolitis in larvae increased the proportion of *Proteobacteria* (especially *Burkholderia*) and decreased the relative number of Firmicutes (*Lactobacillus* group) in the composition of the larval microbiota, and these changes correlated with the severity of the enterocolitis (He et al., 2013). Cotreatment with excretory-secretory products from the nematode *Anisakis* showed a suppression on TNBS-induced mortality and pro-inflammatory gene expression in adult zebrafish, suggesting that the exposure to the immunomodulatory effects of parasitic helminths could be protective against IBD (Haarder et al., 2017). Recently, it was observed that a specific plasma fatty acid, palmitic acid, exacerbated TNBS-induced enterocolitis in zebrafish larvae, indicating that fatty acids can modulate intestinal inflammation, which is in line with a hypothesis which was derived from a meta-analysis of human genome-wide association studies (Wang et al., 2015).

Validation of the larval TNBS-induced enterocolitis model was further performed using known (steroidal and non-steroidal) anti-inflammatory and antibiotic drug treatments which ameliorated the response to TNBS (Fleming et al., 2010; Oehlers et al., 2011). A small drug screen was performed using this model as well, in which NOS inhibitors and thalidomide and parthenolide were tested. Whereas, thalidomide and parthenolide showed a reduction of TNF-α expression (based on immunohistochemistry), only the NOS inhibitors rescued the *in vivo* disease phenotype, assessed by histological analysis (Fleming et al., 2010). The hyperthermostable superoxide dismutase from *Thermus thermophilus* HB27 was also shown to decrease TNBS-induced intestinal enlargement and neutrophil infiltration (Chuang et al., 2019; Sheng et al., 2019). Similarly, the DSS-induced model was validated by demonstrating the role of retinoic acid (RA) in suppressing the pathological intestinal mucin production (Oehlers et al., 2012). Furthermore, it was reported that prostaglandin E2 was able to rescue the loss of mucus layers and the damage in epithelial barrier due to DSS treatment, thereby providing protection against injury, and that other commonly utilized IBD medications, mesalamine and 6-mercaptopurine, also showed protective effects in the DSS model (Chuang et al., 2019). In addition, the DSS- and TNBS-induced larval enterocolitis models have been used for screening small molecules from a large clinical compound library using the neutrophil accumulation in the intestine as a readout (Oehlers et al., 2017). Most of the hits were known antibiotics or anti-inflammatory agents, confirming the validity of the screening assay. Novel drug hits were also identified using this assay, such as cholecystokinin (CCK) and dopamine receptor agonists, and the involvement of these receptors was confirmed by using CCK and dopamine receptor antagonists, which were shown to exacerbate inflammation in these models (Oehlers et al., 2017).

### Mutation-Induced Inflammation

#### The *hai1a* Mutant

To identify genes with essential functions during zebrafish skin development, a screen of mutants generated by insertional mutagenesis was performed (Amsterdam et al., 1999), and a mutant line was identified carrying an insertion in the hepatocyte growth factor activator inhibitor 1a gene (*hai1a*, also known as *spint1lb*) (Carney et al., 2007; Mathias et al., 2007). Hai1a is known to be an inhibitor of serine proteases, in particular of Matriptase 1a. The *hai1a* mutant zebrafish larvae display a phenotype reminiscent of the human condition psoriasis: the basal keratinocytes in the epidermis lose their regular polygonal shape and the tight contact to adjacent cells, form aggregates and display enhanced apoptosis. These epidermal defects induce an inflammatory response in the skin, which is illustrated by leukocytes strongly accumulating near aggregates of keratinocytes with apoptotic cells at 1 dpf (Carney et al., 2007; Mathias et al., 2007). The mutant neutrophils display a more random motility, but retain their ability to respond to directional signals (Mathias et al., 2007). A microarray transcriptome analysis showed that the expression of pro-inflammatory genes was increased in the mutant fish (LeBert et al., 2015). Among those genes, matrix metalloproteinase 9 gene (*mmp9*) played a critical role. Morpholino knockdown of *mmp9* partially rescued the abnormal epithelial phenotype as well as the neutrophilic infiltration of the epithelium, and restored the organization of collagen fibers.

#### The *cdipt* Mutant

Screening the same collection of insertional mutants, in which the *hai1a* mutant was found (Amsterdam et al., 1999), for liver defects, a mutant with an insertion in the cdp-diacylglycerolinositol 3-phosphatidyltransferase (*cdipt)* gene was identified (Thakur et al., 2011). Cdipt, also known as Phosphatidylinositol synthase, has an indispensable role in the synthesis of a critical phospholipid, phosphatidylinositol (PtdIns). The mutant larvae displayed chronic endoplasmic reticulum (ER) stress which contributes to hepatic steatosis around 5 dpf, resembling features of non-alcoholic fatty liver disease in humans (Thakur et al., 2011). A mild inflammatory response was observed, reflected by the presence of macrophages adjacent to necrotic hepatocytes and increased expression of inflammatory genes. More recently, it was reported that the *cdipt* mutant shows a pathological phenotype in the gastro-intestinal tract reminiscent of IBD (Thakur et al., 2014). The PtdIns deficiency led to an ER stress-mediated cytopathology in intestinal epithelial cells, including vacuolation, microvillus atrophy and impaired proliferation, subsequently resulting in reduced mucus secretion, goblet cell apoptosis, autophagy, and bacterial overgrowth. Eventually, this results in an inflammatory response, reflected by the infiltration of macrophages and neutrophils into the intestines. The inflammation could be suppressed by antibiotics and anti-inflammatory drugs, but these treatments failed to suppress the ER stress phenotype. Treatment of mutant larvae with phenylbutyric acid (PBA), a small chemical chaperone and a well-established drug proven to reduce ER stress, was shown to alleviate the mutant phenotype (Thakur et al., 2014).

## The Use of Zebrafish Inflammation Models for Research on Glucocorticoid Drugs

Steroidal anti-inflammatory drugs, also referred to as GCs, have been studied extensively using zebrafish inflammation models. This research has focused on the molecular mechanisms underlying the anti-inflammatory action of these compounds and aims at the development of novel GC drugs. In addition, due to their well-characterized anti-inflammatory effects, GCs are frequently used as a positive control in anti-inflammatory drug screens and the golden standard for anti-inflammatory drugs, and therefore provide a useful method for validation of novel animal models for inflammation.

GCs are a class of steroid hormones secreted by the adrenal gland, regulating a wide variety of systems in the body, like the immune, metabolic, reproductive, cardiovascular and central nervous system (Chrousos and Kino, 2005; Revollo and Cidlowski, 2009; Oakley and Cidlowski, 2013). In humans, the secretion of the main endogenous GC, cortisol, shows a diurnal pattern, is greatly enhanced upon stress, and is mainly regulated by the hypothalamic-pituitary-adrenal (HPA) axis (Tsigos and Chrousos, 2002; Wei et al., 2017). The immune-suppressive effects of GCs were first reported by Hench et al. (1949), who demonstrated that adrenocorticotropic hormone (ACTH) and cortisone improved clinical features of rheumatoid arthritis patients (Hench et al., 1949). Subsequently, GCs were soon applied in eye inflammation (Duke-Elder and Ashton, 1951; O'Rourke et al., 1956), and currently GCs are frequently prescribed worldwide to treat various immune-related diseases, including asthma, rheumatoid arthritis, dermatitis, leukemia, several autoimmune diseases, and even some cancers, due to their potent and well-established anti-inflammatory and immune-suppressive effects (Barnes, 2011; Busillo and Cidlowski, 2013). These effects of GCs are mediated by an intracellular receptor, the glucocorticoid receptor (GR). GCs activate the translocation of this receptor from the cytoplasm to the nucleus, where it acts as a transcription factor, inducing the expression of anti-inflammatory genes and inhibiting the transcriptional activity of pro-inflammatory genes (Baschant and Tuckermann, 2010; Busillo and Cidlowski, 2013).

Like in humans, the main endogenous GC hormone in fish is cortisol and its secretion is regulated by the hypothalamus-pituitary-interrenal (HPI) axis, the fish equivalent of the HPA axis (Wendelaar Bonga, 1997; Schreck et al., 2016). Zebrafish, similarly to humans, have a Gr that is encoded by a single *gr* gene (Alsop and Vijayan, 2008; Schaaf et al., 2008). In addition, both zebrafish and humans express an alternative splice variant, Grβ, which is notably absent in mice (Schaaf et al., 2008). The zebrafish Gris structurally and functionally highly similar to its mammalian equivalent, which includes the immune-suppressive action that is observed upon Gr activation in zebrafish (Schaaf et al., 2008, 2009).

Upon tail amputation in embryos, treatment with several synthetic GCs has been shown to inhibit the migration of neutrophils toward the wounded site in a Gr-dependent manner. However, GCs leave the migration of macrophages unaffected in most studies (Mathew et al., 2007; Zhang et al., 2008; Hall et al., 2014a; Chatzopoulou et al., 2016; Xie et al., 2019). However, in some studies an effect of dexamethasone on macrophage migration has been reported as well (Cholan et al., 2020). What causes this discrepancy is still unclear, but it may be related to specific (non-Gr-mediated) effects of this glucocorticoid drug or the use of a different transgenic line. The Gr-induced upregulation of the expression of the gene encoding MAPK phosphatase-1 (Mkp-1) was suggested to be involved in the inhibition of neutrophil migration, by inactivation of JNK, resulting in a reduced AP-1-induced transcriptional activation of pro-inflammatory genes (Zhang et al., 2008). Indeed, studying the transcriptome by microarray analysis showed that almost all wounding-induced changes in transcription were attenuated by GC treatment (Chatzopoulou et al., 2016). Although the chemotactic migration of macrophages is not affected by GCs, their differentiation toward a pro-inflammatory (M1) phenotype is inhibited upon GC treatment (Xie et al., 2019). In a combined infection/tail wounding model, GCs were shown to inhibit the infection-induced expression in epidermal and/or epidermal cells of *irg1l*, thereby inhibiting the ROS production which is important for leukocyte migration (Hall et al., 2014a). In adult zebrafish, no effect of GC treatment on neutrophil recruitment upon tail wounding was detected (Geurtzen et al., 2017). In adult zebrafish models for brain and heart injuries, GCs were shown to inhibit the expression of pro-inflammatory genes like *il8, tnfa*, and *il1b* and reduced the recruitment of leukocytes toward the wounded area (Kyritsis et al., 2012; Huang et al., 2013).

In the LPS-induced inflammation model, GC administration was reported to inhibit the production of ROS and NO, the expression of pro-inflammatory genes, the recruitment of leukocytes, and the mortality (Wijesinghe et al., 2014; Yang et al., 2014, 2017; Sun et al., 2017). In the Copper-induced inflammation model using CuSO_4_ immersion of larvae, GCs also caused inhibition of neutrophil accumulation (d'Alençon et al., 2010). Similarly, utilizing the DSS-induced enterocolitis model, GCs were observed to inhibit the expression of pro-inflammatory genes and neutrophil infiltration (Oehlers et al., 2012). Interestingly, in larvae from a CRISP/Cas9-generated Gr mutant line, the DSS-induced increase in pro-inflammatory gene expression was abolished due to the deficiency in Gr signaling, suggesting a dual action, both pro- and anti-inflammatory, of GC signaling in the immune system (Facchinello et al., 2017).

The clinical use of GCs is severely limited by the severity of their side effects, which include diabetes and obesity, osteoporosis, and impaired wound healing. Interestingly, these effects have been modeled in zebrafish as well, opening up the possibility to evaluate both the therapeutic anti-inflammatory effect and the adverse effects. GC effects on metabolism, including increased glucose concentrations, were observed in zebrafish embryos and the global transcriptional changes underlying these effects have been characterized (Chatzopoulou et al., 2015). GC-induced osteoporosis was modeled by treating larvae with GCs between 5 and 10 dpf and performing staining with alizarin red (which binds to calcified matrix) (Barrett et al., 2006), and studying extracellular matrix (ECM)-, osteoblast-, and osteoclast-related genes (He et al., 2018; Huo et al., 2018). Alternatively, regenerating scales that were removed from GC-treated adult fish have been used to model GC-induced osteoporosis (de Vrieze et al., 2014). Finally, inhibitory effects on tissue regeneration and wound healing have been shown in many zebrafish injury models. Inhibition of regeneration by GCs was observed after spinal motor neuron lesions in larvae (Ohnmacht et al., 2016), and in adult zebrafish after tail fin amputation, brain lesion, and cardiac injury, GCs were demonstrated to inhibit tissue regeneration (Kyritsis et al., 2012; Huang et al., 2013; Geurtzen et al., 2017; Geurtzen and Knopf, 2018). GC treatment of zebrafish embryos blocks the regeneration of the tail fin upon amputation through inhibition on blastemal formation and cell proliferation (Mathew et al., 2007; Sengupta et al., 2012). Interestingly, the ginsenoside Rg1 was shown to inhibit neutrophil migration in a Gr-dependent manner, but did not show any effect on tissue regeneration. These data suggest that this compound may be provide an interesting lead for the development of novel anti-inflammatory drugs with reduced side effects (He et al., 2020).

## Concluding Remarks

The use of animal models is a critical part of biomedical research and crucial for the development of novel drugs. A wide range of human disease models have been established in mammalian models such as rats and mice, which have largely contributed to the remarkable progress in our understanding of the mechanisms underlying these diseases and the development of novel therapies. However, the rodent systems have limitations such as the high cost of housing and breeding and they are not suited for large-scale automated screening. The development of the zebrafish animal model in the past decades has added a complementary system, which allows the performance of automated high through-put screening *in vivo*, mainly due to the small size and transparency of zebrafish larvae. The similarities of the immune system and inflammatory responses between zebrafish and mammals guarantee good translational value.

In order to model inflammatory diseases, three types of inflammation models have been developed in zebrafish: wounding-, chemical-, and mutation-induced inflammation. These models have enabled a detailed investigation of the cellular and molecular mechanisms underlying the inflammatory response, adding to our knowledge of the mechanisms of leukocyte behavior and the identification of potential drug targets. For example, using the zebrafish model, it was observed for the first time that a tissue-scale H_2_O_2_ gradient is created during the onset of an inflammatory response which signals to leukocytes in the tissues (Niethammer et al., 2009), and that Lyn acts as a redox sensor to mediate the migration of leukocyte (Yoo et al., 2011). In addition, the described models have been used for the screening of compound libraries. This has led to the discovery of important novel targets for anti-inflammatory drugs, such as ErbBs (Rahman et al., 2019). Moreover, various drug candidates were tested or identified, such as natural extracts [e.g., fucoidan (Lee et al., 2013), tashinone IIA (Robertson et al., 2014), and cymopol (Bousquet et al., 2020)], thalidomide analogs (Beedie et al., 2016), and the PI3K inhibitor LY294002 (Bischel et al., 2013). In summary, these zebrafish inflammation models have been shown to be very useful to unravel the molecular and cellular aspects of the inflammatory response and for the discovery of novel drug targets. Besides, these models have proven to be effective screening tools for candidate drugs, providing an intermediate between *in vitro* assays and rodent experiments with great potential to accelerate the preclinical phase of anti-inflammatory drug development.

## Author Contributions

AM and MS: conceptualization, resources, and supervisions. YX, AM, and MS: methodology and writing–review & editing. YX: investigation and funding acquisition. YX and MS: data curation and writing–original draft. MS: project administration. All authors contributed to the article and approved the submitted version.

## Conflict of Interest

The authors declare that the research was conducted in the absence of any commercial or financial relationships that could be construed as a potential conflict of interest.
